# Crystal structure of (2*E*)-1-(4-eth­oxy­phen­yl)-3-(4-fluoro­phen­yl)prop-2-en-1-one

**DOI:** 10.1107/S2056989022007423

**Published:** 2022-07-22

**Authors:** Merle Bernhard, Jacob C. Lutter, Allison Predecki

**Affiliations:** a Department of Chemistry and Biochemistry, Shippensburg University, Shippensburg, PA 17257, USA; b Department of Chemistry and Biochemistry, University of Southern Indiana, Evansville, IN 47712, USA; Purdue University, USA

**Keywords:** crystal structure, chalcones, 4 and 4′ substitution, conjugated torsions

## Abstract

The title mol­ecule, C_17_H_15_FO_2_, was synthesized by a Claisen–Schmidt condensation with 4-fluoro­benzaldehyde and 4′-eth­oxy­aceto­phenone. The torsion angles between the 4-fluoro­phenyl ring and the alkene and the 4′-eth­oxy­phenyl ring and the 2-propen-1-one are −1.2 (4) and 1.2 (3)°, respectively; however, there is a larger torsion between the bonds comprising the 2-propen-1-one unit of 12.0 (4)°. The crystal packing is stabilized by inter­molecular C—H⋯O/F hydrogen bonding, π–π stacking, and H–π inter­actions.

## Abstract 2

The title mol­ecule is nearly planar with a slight structural bend involving the carbonyl group. The mol­ecules pack in the crystal by inter­molecular C—H⋯O/F hydrogen bonding, π–π stacking, and H–π inter­actions.

## 1. Chemical context

Chalcones are a group of 1,3-diaryl-2-propen-1-one compounds that have been found to exhibit a wide variety of biological activity including anti­cancer, anti­microbial and anti-inflammatory properties (Sahu *et al.*, 2012 ▸). Chalcones are also important starting materials for the synthesis of several pharmacologically inter­esting classes of heterocyclic compounds such as isoxazoles, pyrazolines and pyrazoles (Kamal *et al.*, 2019 ▸). In our research involving the synthesis of chalcone derivatives, we have synthesized and obtained an X-ray structure for the title compound, C_17_H_15_FO_2_, 2(*E*)-1-(4-eth­oxy­phen­yl)-3-(4-fluoro­phen­yl)-2-propen-1-one. 
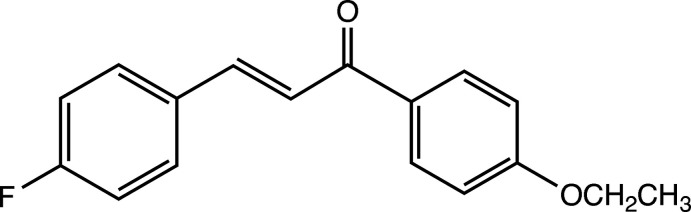


## 2. Structural commentary

**Figure 1:**
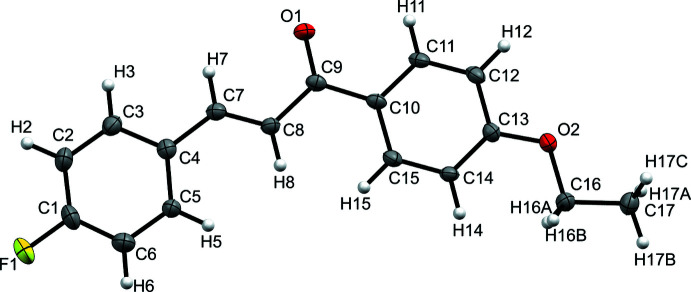
The mol­ecular structure of 2(*E*)-1-(4-eth­oxy­phen­yl)-3-(4-fluoro­phen­yl)-2-propen-1-one. Displacement ellipsoids are drawn at the 50% probability level.

This chalcone has aromatic rings with substitutions in the 4 position on both ends of the mol­ecule, where the phenyl on the alkene is fluorinated, and the phenyl on the carbonyl contains an ethoxide (Fig. 1 ▸). Both phenyl rings are inclined towards the same side of the mol­ecule thanks to the *E* geometry of the chalcone’s alkene. The compound is a heavily π-conjugated structure that is nearly planar. To measure the deviation from planarity, three torsion angles were examined. The angles involving the aromatic rings are nearly identical with little bend, where the torsion between the C8—C7 and C5—C4 bonds is −1.2 (4)°, and the torsion between the C8—C9 and C15—C10 bonds is 1.2 (3)°. However, the torsion angle of the chalcone between the O1—C9 and C7—C8 bonds is 12.0 (4)°, indicating a break in planarity. This single deviation causes a slight concave bend in the mol­ecule. The title compound crystallized as a racemic mixture in the space group *Pca*2_1_; thus, a clockwise and anti­clockwise torsion of the chalcone are present with a 1:1 ratio in the unit cell.

There are several other chalcones with a comparable 4 and 4′ set of substitutions that are summarized in Table 1 ▸ from a CSD database search. If the halogen (–*X*) is maintained as a fluorine, the other substituent (–*R*) varies as either a methyl, hydroxyl, meth­oxy, or eth­oxy group. Examination of the three torsion angles described above suggests that there is a trend in the degree of distortion from planarity, with an order of methyl, meth­oxy, eth­oxy, to hy­droxy by increasing planarity. While there are no direct examples that contain a halogen and an eth­oxy, we felt comparison of our compound to the nearest chloro- and bromo-substituted compounds was warranted. The closest examples are a bromo/meth­oxy and a chloro/meth­oxy 4,4′ -substituted chalcone. Both cases are more distorted from planar than our fluoro/eth­oxy chalcone. Lastly, we found a set of chalcones with an eth­oxy substituent, where there are chlorine atoms in the 2 and 3 position of the respective phenyl ring. Both of these cases are more planar than our chalcone.

## 3. Supra­molecular features

**Figure 2:**
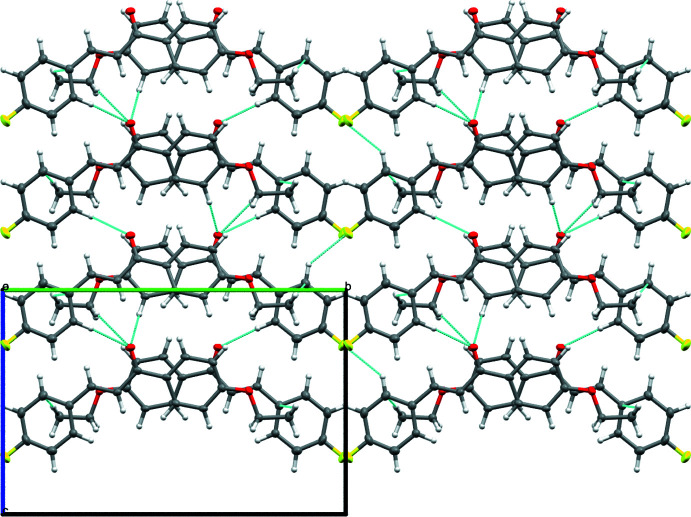
Packing of the title compound viewed along the *a* axis. Hydrogen bonds and H–π bonds are shown as blue lines.

2(*E*)-1-(4-Eth­oxy­phen­yl)-3-(4-fluoro­phen­yl)-2-propen-1-one crystallizes in the ortho­rhom­bic space group *Pca*2_1_, with four mol­ecules occupying one unit cell. The mol­ecules pack using hydrogen bonding, π–π stacking, and H–π inter­actions (Figs. 2 ▸, 3 ▸, 4 ▸). There are four hydrogen bonds (Table 2 ▸) that inter­connect each mol­ecule to three of its neighbors. The first is between the C3—H3 bond and an adjacent F1 atom, the second pairs the C5—H5 bond and a nearby O1 atom, and the final two involve the C14—H14 and C16—H16*A* bonds with a neighboring O1 atom. Given the extent of conjugated π bonds throughout this mol­ecule, π–π stacking is present between adjacent mol­ecules along the *a* axis [centroid–centroid distance = 4.240 Å], with alternating mol­ecules related by the *a* glide plane of the *Pca*2_1_ space group; this orients these mol­ecules such that adjacent mol­ecules are mirror images of one another with opposing chalcone bond torsions. Lastly, there are H–π inter­actions present between H17*A* and the aromatic ring comprised of C1–C6, as well as between H17*C* and this same ring on another mol­ecule, forming a chain of inter­actions that parallel the *a* axis.

In comparison to the other chalcones described in Table 1 ▸, our structure packs in a unique space group *Pca*2_1_, where many others pack in *Pbca* or *P* 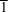 . Common themes that appear among these structures include π–π stacking and hydrogen bonding to the carbonyl oxygen. However, it is inter­esting to note that the chloro/meth­oxy and bromo/meth­oxy analogs pack with π–π stacking where the mol­ecules are mirror images from a plane that is colinear with the mol­ecular mean plane, rather than images related to a plane that is orthogonal to the mol­ecular mean plane as observed in our structure. Also, in the case where the –*R* substituent is a hydroxide, hydrogen bonding between the hydroxyl group and and the carbonyl oxygen dominates the packing.

## 4. Database survey

A search of the Cambridge Structural Database (CSD, Version 5.43, update March 2022; Groom *et al.*, 2016 ▸) for 4,4′-phenyl-substituted chalcones resulted in multiple hits. Most closely related to the title compound are three 4-fluoro­phenyl-substituted chalcones: (*E*)-3-(4-fluoro­phen­yl)-1-(4-methyl­phen­yl)prop-2-en-1-one (Butcher *et al.*, 2007 ▸), (*E*)-3-(4-fluoro­phen­yl)-1-(4-hydro­yxlphen­yl)prop-2-en-1-one (Sobolev *et al.*, 2022 ▸), (*E*)-3-(4-fluoro­phen­yl)-1-(4-meth­oxy­lphen­yl)prop-2-en-1-one (Zhao *et al.*, 2009 ▸). Additionally, two 4′-meth­oxy-susbstituted compounds with 4-chloro or bromo­phenyl substitution were found, (*E*)-3-(4-chloro­phen­yl)-1-(4-meth­oxy­phen­yl)prop-2-en-1-one (Whitwood *et al.*, 2021 ▸) and (*E*)-3-(4-bromo­phen­yl)-1-(4-meth­oxy­phen­yl)prop-2-en-1-one (Wilhelm *et al.*, 2022 ▸). Two 4′-eth­oxy-substituted compounds were also found, (*E*)-3-(2-chloro­phen­yl)-1-(4-eth­oxy­lphen­yl)prop-2-en-1-one (Harshitha *et al.*, 2018 ▸) and (*E*)-3-(3-chloro­phen­yl)-1-(4-eth­oxy­lphen­yl)prop-2-en-1-one (Harsh­itha *et al.*, 2018 ▸). See Table 1 ▸ for relevant data from these structures.

## 5. Synthesis and crystallization

4-Fluoro­benzaldehyde (3 mmol) and 4-eth­oxy­aceto­phenone (3 mmol) were mixed in 95% EtOH (2.5 mL). An aqueous solution of sodium hydroxide (0.3 mL, 15 m*M*) was added to the mixture dropwise. The mixture was allowed to stir at room temperature for 45 min. Cold distilled H_2_O (4 mL) was added and the mixture was cooled in an ice bath before isolating the solid product by vacuum filtration. The chalcone was purified by recrystallization with di­chloro­methane/hexane (4:1) to yield colorless crystals (77% yield). High-quality crystals for diffraction were grown from slow evaporation of 190 proof ethanol at room temperature, m.p. 392–394 K; IR (ATR) *ν*_max_ 3067, 2936, 1653, 1596, 1572, 1504, 1157, 1033 cm^−1^; ^1^H NMR (400 MHz, CDCl_3_) *δ* 8.03 (*d*, *J* = 9.2 Hz, 2H), 7.76 (*d*, *J* = 15.6 Hz, 1H), 7.62 (*dd*, *J* = 5.5, 3.2 Hz, 2H), 7.46 (*d*, *J* = 15.6 Hz, 1H), 7.10 (*t*, *J* = 8.7 Hz, 2H), 6.79 (*d*, *J* = 8.7 Hz, 2H), 3.88 (*s*, 3H) ppm; ^13^C{^1^H} NMR (100MHz, CDCl_3_) *δ* 188.4, 163.5, 163.9 (*d*, ^1^ *J*_C–F_ = 252.1 Hz), 142.6, 131.3 (*d*, ^4^ *J*_C–F_ = 2.9 Hz), 131.0, 130.8, 130.2 (*d*, ^3^ *J*_C–F_ = 8.6 Hz), 121.5 (*d*, ^6^ *J*_C–F_ = 1.9 Hz), 116.0 (*d*, ^2^ *J*_C–F_ = 22.0 Hz), 113.8, 55.5 ppm. ^1^H NMR data have previously been reported (Liu *et al.*, 2001 ▸).

## 6. Refinement

Crystal data, data collection and structure refinement details are summarized in Table 3 ▸. Hydrogen atoms were generated using a riding model with geometric constraints and refined isotropically. Aromatic C—H distances are 0.95 Å, methyl­ene C—H distances are 0.99 Å, and methyl C—H distances are 0.98 Å. *U*_iso_(H) was 1.2 times *U*_eq_(C) for aromatic and methyl­ene hydrogen atoms, and 1.5 times *U*_eq_(C) for methyl hydrogen atoms. There is minor whole mol­ecule disorder visible in the residual peaks that was not refined since these peaks are rather small (< 0.26 e Å^−3^) and there was little improvement in the model.

## Supplementary Material

Crystal structure: contains datablock(s) I. DOI: 10.1107/S2056989022007423/zl5033sup1.cif

Structure factors: contains datablock(s) I. DOI: 10.1107/S2056989022007423/zl5033Isup2.hkl

Supporting information file. DOI: 10.1107/S2056989022007423/zl5033Isup3.cml

CCDC reference: 2191384

Additional supporting information:  crystallographic information; 3D view; checkCIF report

## Figures and Tables

**Figure 3:**
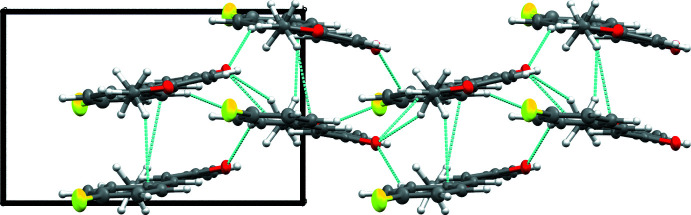
Packing of the title compound viewed along the *b* axis. Hydrogen bonds and H–π bonds are shown as blue lines.

**Figure 4:**
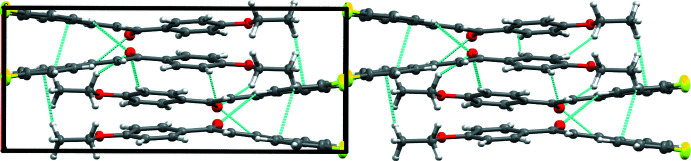
Packing of the title compound viewed along the *c* axis. Hydrogen bonds and H–π bonds are shown as blue lines.

**Table 1:** Torsions of 4,4′ substituted chalcones (°) All torsions were measured in *Mercury* (v2020.2.0; Macrae *et al.*, 2020 ▸), with the exception of the torsion angles from this work, which were calculated using the CONF command of *SHELXL2018/3* (Sheldrick, 2015*b* ▸).

Entry	–*X*	–*R*	Carbon­yl–alkene torsion	Ar­yl–alkene torsion	Ar­yl–carbonyl torsion	Space group	CCDC Dep. No.
1	F	Me	18.77	12.69	13.71	*P*2_1_/*c*	660304*^*a*^*
2	F	OH	2.41	0.99	6.18	*P* 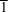	2184323*^*b*^*
3	F	OMe	18.95	11.50	2.92	*Pbca*	738291*^*c*^*
4	F	OEt	12.00	1.20	1.20	*Pca*2_1_	This work
5	Cl	OMe	16.38	3.78	25.77	*Pbca*	2070477*^*d*^*
6	Br	OMe	16.42	6.43	24.69	*Pc*	2062759*^*e*^*
7	2-Cl	OEt	5.16	4.47	0.44	*P* 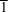	1550212*^*f*^*
8	3-Cl	OEt	0.86	0.93	2.60	*P* 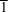	1587066*^*f*^*

Notes: (*a*) Butcher *et al.* (2007 ▸); (*b*) Sobolev *et al.* (2022 ▸); (*c*) Zhao *et al.* (2009 ▸); (*d*) Whitwood *et al.* (2021 ▸); (*e*) Wilhelm *et al.* (2022 ▸); (*f*) Harshitha *et al.* (2018 ▸).

**Table 2:** Hydrogen-bond geometry (Å, °) Two hydrogen bonds were found automatically by *SHELXL*; including the C14—H14 and O1, and C16—H16*A* and O1 donor–acceptor pairs. The remaining two pairs were identified by inspection.

*D*—H⋯*A*	*D*—H	H⋯*A*	*D*⋯*A*	*D*—H⋯*A*
C3—H3⋯F1^i^	0.95	2.60	3.345 (3)	136
C5—H5⋯O1^ii^	0.95	2.70	3.544 (3)	149
C14—H14⋯O1^iii^	0.95	2.46	3.295 (3)	146
C16—H16*A*⋯O1^iii^	0.99	2.60	3.470 (3)	146

Symmetry codes: (i) 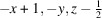 ; (ii) 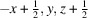 ; (iii) 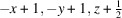 .

**Table 3:** Experimental details

Crystal data	
Chemical formula	C_17_H_15_FO_2_
*M*_r_	270.29
Crystal system, space group	Orthorhombic, *P* *c* *a*2_1_
Temperature (K)	100
*a*, *b*, *c* (Å)	7.1426 (4), 17.0566 (9), 11.1520 (6)
*V* (Å^3^)	1358.63 (13)
*Z*	4
Radiation type	Mo *K*α
μ (mm^−1^)	0.10
Crystal size (mm)	0.24 × 0.11 × 0.11

Data collection	
Diffractometer	Bruker APEXII CCD
Absorption correction	Multi-scan (*SADABS*; Krause *et al.*, 2015 ▸)’
*T*_min_, *T*_max_	0.691, 0.745
No. of measured, independent and observed [*I* > 2σ(*I*)] reflections	23454, 2438, 2365
*R*_int_	0.061
(sin θ/λ)_max_ (Å^−1^)	0.603

Refinement	
*R*[*F* ^2^ > 2σ(*F* ^2^)], *wR*(*F* ^2^), *S*	0.039, 0.104, 1.10
No. of reflections	2438
No. of parameters	182
No. of restraints	1
H-atom treatment	H-atom parameters constrained
Δρ_max_, Δρ_min_ (e Å^−3^)	0.27, −0.17

Computer programs: *APEX4* (Bruker, 2021 ▸), *SAINT* (Bruker, 2002 ▸), *SHELXT2018/2* (Sheldrick, 2015*a* ▸), *SHELXL2018/3* (Sheldrick, 2015*b* ▸), and *SHELXTL* (Sheldrick, 2008 ▸).

## supplementary crystallographic information

### Crystal data

**Table:**

C_17_H_15_FO_2_	*D*_x_ = 1.321 Mg m^−^^3^
*M**_r_* = 270.29	Melting point = 119–121 K
Orthorhombic, *P**c**a*2_1_	Mo *K*α radiation, λ = 0.71073 Å
*a* = 7.1426 (4) Å	Cell parameters from 7611 reflections
*b* = 17.0566 (9) Å	θ = 3.1–25.3°
*c* = 11.1520 (6) Å	µ = 0.10 mm^−^^1^
*V* = 1358.63 (13) Å^3^	*T* = 100 K
*Z* = 4	Block, colorless
*F*(000) = 568	0.24 × 0.11 × 0.11 mm

### Data collection

**Table:**

Bruker APEXII CCD diffractometer	2365 reflections with *I* > 2σ(*I*)
φ and ω scans	*R*_int_ = 0.061
Absorption correction: multi-scan (SADABS; Krause *et al.*, 2015)'	θ_max_ = 25.4°, θ_min_ = 2.4°
*T*_min_ = 0.691, *T*_max_ = 0.745	*h* = −8→8
23454 measured reflections	*k* = −20→20
2438 independent reflections	*l* = −13→13

### Refinement

**Table:**

Refinement on *F*^2^	Primary atom site location: dual
Least-squares matrix: full	Secondary atom site location: difference Fourier map
*R*[*F*^2^ > 2σ(*F*^2^)] = 0.039	Hydrogen site location: inferred from neighbouring sites
*wR*(*F*^2^) = 0.104	H-atom parameters constrained
*S* = 1.10	*w* = 1/[σ^2^(*F*_o_^2^) + (0.069*P*)^2^ + 0.1828*P*] where *P* = (*F*_o_^2^ + 2*F*_c_^2^)/3
2438 reflections	(Δ/σ)_max_ < 0.001
182 parameters	Δρ_max_ = 0.27 e Å^−^^3^
1 restraint	Δρ_min_ = −0.17 e Å^−^^3^

### Special details

**Table:**

Geometry. All esds (except the esd in the dihedral angle between two l.s. planes) are estimated using the full covariance matrix. The cell esds are taken into account individually in the estimation of esds in distances, angles and torsion angles; correlations between esds in cell parameters are only used when they are defined by crystal symmetry. An approximate (isotropic) treatment of cell esds is used for estimating esds involving l.s. planes.
Refinement. There is a minor disordered component visible in the residual peaks that is not refined, since there is little improvement in the model.

### Fractional atomic coordinates and isotropic or equivalent isotropic displacement parameters (Å^2^)

**Table:**

	*x*	*y*	*z*	*U*_iso_*/*U*_eq_	
F1	0.4847 (3)	0.00701 (11)	0.7445 (2)	0.0476 (6)	
O1	0.3123 (3)	0.37163 (10)	0.25635 (16)	0.0242 (4)	
O2	0.3964 (2)	0.71755 (10)	0.44972 (18)	0.0227 (4)	
C1	0.4610 (4)	0.06802 (16)	0.6686 (3)	0.0326 (7)	
C2	0.4545 (4)	0.05380 (16)	0.5477 (3)	0.0301 (6)	
H2	0.465389	0.001957	0.517328	0.036*	
C3	0.4316 (4)	0.11687 (16)	0.4708 (3)	0.0257 (6)	
H3	0.426726	0.107993	0.386721	0.031*	
C4	0.4154 (3)	0.19357 (15)	0.5148 (2)	0.0205 (6)	
C5	0.4225 (4)	0.20530 (15)	0.6394 (2)	0.0220 (6)	
H5	0.410714	0.256768	0.671075	0.026*	
C6	0.4464 (4)	0.14242 (17)	0.7165 (3)	0.0301 (6)	
H6	0.452542	0.150326	0.800692	0.036*	
C7	0.3907 (3)	0.25841 (15)	0.4307 (2)	0.0208 (6)	
H7	0.390057	0.244965	0.348020	0.025*	
C8	0.3692 (4)	0.33370 (14)	0.4560 (2)	0.0199 (5)	
H8	0.369696	0.350200	0.537401	0.024*	
C9	0.3442 (3)	0.39260 (15)	0.3600 (2)	0.0185 (5)	
C10	0.3596 (3)	0.47712 (15)	0.3897 (2)	0.0179 (5)	
C11	0.3348 (4)	0.53263 (15)	0.2981 (2)	0.0211 (5)	
H11	0.309041	0.515191	0.218899	0.025*	
C12	0.3470 (3)	0.61131 (15)	0.3206 (2)	0.0225 (6)	
H12	0.328745	0.647740	0.257190	0.027*	
C13	0.3862 (3)	0.63855 (14)	0.4366 (2)	0.0195 (6)	
C14	0.4132 (3)	0.58453 (15)	0.5286 (2)	0.0192 (5)	
H14	0.440382	0.602146	0.607528	0.023*	
C15	0.4002 (4)	0.50479 (15)	0.5046 (2)	0.0198 (5)	
H15	0.419362	0.468300	0.567816	0.024*	
C16	0.4192 (4)	0.74662 (15)	0.5705 (3)	0.0236 (6)	
H16A	0.536921	0.726316	0.605590	0.028*	
H16B	0.313446	0.729185	0.621292	0.028*	
C17	0.4247 (4)	0.83493 (15)	0.5646 (3)	0.0264 (6)	
H17A	0.540214	0.851724	0.524786	0.040*	
H17B	0.420936	0.856464	0.646047	0.040*	
H17C	0.316396	0.853991	0.519179	0.040*	

### Atomic displacement parameters (Å^2^)

**Table:**

	*U*^11^	*U*^22^	*U*^33^	*U*^12^	*U*^13^	*U*^23^
F1	0.0811 (15)	0.0254 (9)	0.0364 (10)	0.0047 (9)	−0.0034 (10)	0.0135 (8)
O1	0.0317 (10)	0.0254 (9)	0.0156 (9)	−0.0012 (7)	0.0005 (8)	−0.0020 (8)
O2	0.0303 (10)	0.0174 (9)	0.0204 (10)	−0.0012 (7)	−0.0030 (8)	0.0031 (7)
C1	0.0432 (18)	0.0207 (14)	0.0338 (18)	0.0018 (12)	−0.0004 (13)	0.0099 (13)
C2	0.0397 (15)	0.0172 (12)	0.0334 (16)	0.0016 (11)	0.0008 (13)	−0.0019 (12)
C3	0.0283 (14)	0.0233 (13)	0.0256 (15)	0.0002 (11)	−0.0012 (11)	−0.0040 (11)
C4	0.0185 (12)	0.0206 (12)	0.0225 (14)	−0.0004 (9)	−0.0001 (10)	0.0007 (11)
C5	0.0281 (14)	0.0191 (13)	0.0190 (13)	0.0004 (10)	0.0017 (11)	0.0002 (10)
C6	0.0391 (16)	0.0305 (15)	0.0208 (14)	0.0000 (12)	0.0007 (12)	0.0009 (12)
C7	0.0221 (12)	0.0237 (13)	0.0166 (13)	−0.0011 (10)	0.0004 (10)	−0.0002 (11)
C8	0.0234 (11)	0.0211 (12)	0.0153 (12)	−0.0009 (10)	0.0014 (10)	−0.0016 (11)
C9	0.0157 (11)	0.0237 (13)	0.0161 (13)	−0.0003 (9)	0.0031 (9)	0.0015 (10)
C10	0.0160 (11)	0.0244 (13)	0.0134 (12)	−0.0005 (9)	0.0025 (9)	0.0022 (10)
C11	0.0234 (13)	0.0275 (13)	0.0125 (12)	−0.0021 (10)	−0.0020 (10)	0.0023 (10)
C12	0.0257 (14)	0.0239 (13)	0.0181 (14)	−0.0019 (10)	−0.0040 (11)	0.0060 (11)
C13	0.0191 (11)	0.0193 (12)	0.0200 (14)	0.0001 (9)	0.0010 (10)	0.0021 (11)
C14	0.0227 (13)	0.0227 (13)	0.0121 (12)	0.0010 (10)	0.0000 (10)	0.0002 (10)
C15	0.0215 (12)	0.0222 (13)	0.0158 (13)	0.0020 (10)	−0.0013 (10)	0.0037 (10)
C16	0.0306 (13)	0.0200 (13)	0.0202 (13)	−0.0007 (10)	0.0029 (11)	−0.0006 (11)
C17	0.0287 (13)	0.0204 (13)	0.0301 (15)	0.0000 (10)	0.0022 (12)	−0.0021 (12)

### Geometric parameters (Å, º)

**Table:**

F1—C1	1.352 (3)	C8—H8	0.9500
O1—C9	1.231 (3)	C9—C10	1.483 (3)
O2—C13	1.357 (3)	C10—C15	1.396 (4)
O2—C16	1.444 (3)	C10—C11	1.404 (3)
C1—C2	1.371 (5)	C11—C12	1.368 (4)
C1—C6	1.381 (4)	C11—H11	0.9500
C2—C3	1.385 (4)	C12—C13	1.404 (4)
C2—H2	0.9500	C12—H12	0.9500
C3—C4	1.402 (4)	C13—C14	1.392 (3)
C3—H3	0.9500	C14—C15	1.389 (4)
C4—C5	1.405 (4)	C14—H14	0.9500
C4—C7	1.461 (4)	C15—H15	0.9500
C5—C6	1.385 (4)	C16—C17	1.508 (4)
C5—H5	0.9500	C16—H16A	0.9900
C6—H6	0.9500	C16—H16B	0.9900
C7—C8	1.324 (3)	C17—H17A	0.9800
C7—H7	0.9500	C17—H17B	0.9800
C8—C9	1.480 (3)	C17—H17C	0.9800
			
C13—O2—C16	116.6 (2)	C15—C10—C9	123.3 (2)
F1—C1—C2	118.9 (3)	C11—C10—C9	118.9 (2)
F1—C1—C6	118.3 (3)	C12—C11—C10	121.3 (2)
C2—C1—C6	122.7 (3)	C12—C11—H11	119.3
C1—C2—C3	118.4 (3)	C10—C11—H11	119.3
C1—C2—H2	120.8	C11—C12—C13	120.4 (2)
C3—C2—H2	120.8	C11—C12—H12	119.8
C2—C3—C4	121.2 (3)	C13—C12—H12	119.8
C2—C3—H3	119.4	O2—C13—C14	124.8 (2)
C4—C3—H3	119.4	O2—C13—C12	116.0 (2)
C3—C4—C5	118.4 (2)	C14—C13—C12	119.2 (2)
C3—C4—C7	119.4 (3)	C15—C14—C13	119.8 (2)
C5—C4—C7	122.1 (3)	C15—C14—H14	120.1
C6—C5—C4	120.5 (3)	C13—C14—H14	120.1
C6—C5—H5	119.7	C14—C15—C10	121.4 (2)
C4—C5—H5	119.7	C14—C15—H15	119.3
C1—C6—C5	118.8 (3)	C10—C15—H15	119.3
C1—C6—H6	120.6	O2—C16—C17	107.8 (2)
C5—C6—H6	120.6	O2—C16—H16A	110.2
C8—C7—C4	127.7 (3)	C17—C16—H16A	110.2
C8—C7—H7	116.2	O2—C16—H16B	110.2
C4—C7—H7	116.2	C17—C16—H16B	110.2
C7—C8—C9	121.2 (2)	H16A—C16—H16B	108.5
C7—C8—H8	119.4	C16—C17—H17A	109.5
C9—C8—H8	119.4	C16—C17—H17B	109.5
O1—C9—C8	120.3 (2)	H17A—C17—H17B	109.5
O1—C9—C10	120.4 (2)	C16—C17—H17C	109.5
C8—C9—C10	119.3 (2)	H17A—C17—H17C	109.5
C15—C10—C11	117.8 (2)	H17B—C17—H17C	109.5

### Hydrogen-bond geometry (Å, º)

Two hydrogen bonds were found automatically by *SHELXL*; including the C14—H14 and O1, and C16—H16A and O1 donor–acceptor pairs. The remaining two pairs were identified by inspection.

**Table:**

*D*—H···*A*	*D*—H	H···*A*	*D*···*A*	*D*—H···*A*
C3—H3···F1^i^	0.95	2.60	3.345 (3)	136
C5—H5···O1^ii^	0.95	2.70	3.544 (3)	149
C14—H14···O1^iii^	0.95	2.46	3.295 (3)	146
C16—H16*A*···O1^iii^	0.99	2.60	3.470 (3)	146

Symmetry codes: (i) −*x*+1, −*y*, *z*−1/2; (ii) −*x*+1/2, *y*, *z*+1/2; (iii) −*x*+1, −*y*+1, *z*+1/2.
